# Sheep farmer opinions on the current and future role of veterinarians in flock health management on sheep farms: A qualitative study

**DOI:** 10.1016/j.prevetmed.2013.09.009

**Published:** 2013-11-01

**Authors:** Jasmeet Kaler, L.E. Green

**Affiliations:** a The School of Veterinary Medicine and Science, University of Nottingham, Sutton Bonington Campus, Sutton Bonington, Loughborough, Leicestershire, England LE12 5RD, UK; b School of Life Sciences, University of Warwick, Coventry, England CV4 7AL, UK

**Keywords:** Sheep, Farmer opinion, Attitude, Perception, Qualitative, Focus group

## Abstract

A 2009 UK Government report on veterinary expertise in food animal production highlighted that there was insufficient herd health expertise among veterinarians and lack of appropriate business models to deliver veterinary services to the livestock sector. Approximately two thirds of sheep farmers only contact their veterinarian for emergencies and one fifth have all year round contact. The aim of the current study was to understand sheep farmers’ perception, the current and future role of veterinarians in flock health management using qualitative methodology. The eligibility criteria were male farmers with a flock size of at least 200 adult sheep. Seven focus groups of farmers (*n* = 45) stratified by three regions and two age groups (≤50 and >50) were conducted. Thematic analysis of the discussions indicated that most farmers considered and used their veterinarian as a fire-fighter, whilst other advice was gathered free of charge when the veterinarian was on the farm for other reasons (typically seeing cattle) or by telephone. A small group of farmers were using their veterinarian or a sheep consultant proactively with regular contact and found this financially beneficial. Farmers indicated that the key barriers to using a veterinarian proactively were inconsistent service, high turnover of veterinarians, lack of expertise of sheep farming among veterinarians and concern about independence of advice. Although economics was also mentioned as a key barrier to using veterinarians more proactively, most farmers did not know where they gained and lost income from their flock; there was heavy reliance on the single farm payment scheme (SPS) and very few farmers kept records from which they could investigate where there were inefficiencies in production. Overall sheep farmers considered sheep farming complex and that each farm was unique and that they themselves were the experts to manage their flock. We conclude that there is an impasse: veterinarians might need to provide consistency and wide expertise beyond knowledge of disease and a model of how flock planning would be financially beneficial but until sheep farmers keep production records flock health planning cannot be rigorous and the financial benefits cannot be evaluated. Given the reliance on SPS by farmers an alternative model would be to require farmers to keep production records to comply with SPS. This might lead to flock health planning being adopted at a faster rate and so develop the UK sheep industry and make it more environmentally sustainable by reducing waste from disease and low productivity.

## 1 Introduction

Farm animal veterinarians play a key role in the food supply chain, providing expertise in treatment of sick animals and prevention of disease through herd/flock health advisory work. There has been some concern about a shortage of farm animal veterinarians around the world (Bonnet et al., 2011) including in the UK (EFRACom, 2003). The changing role of farm animal veterinarians was the subject of an independent report into veterinarians and veterinary services in the UK (Lowe, 2009). The findings of the report highlighted that there was not an absolute shortage of farm animal veterinarians and that there had been no reduction in their expertise, however, the requirements of producers had changed and veterinarians needed expertise beyond that often currently taught to undergraduate veterinary students which is focused on diagnosis and treatment of sick animals and basic herd management. The Lowe report (2009) stated that farm animal veterinarians needed to sell a differentiated service to meet the demands of current farm businesses. The report omitted to highlight that some veterinarians in the UK had already responded to this changing need and these veterinarians are already providing a wide range of services to some producers, mainly dairy cattle or pig farmers (Dairy Herd Health Group, 2010).

Whilst the Lowe report referred to all farm animal veterinarians, those working with dairy cattle, pigs and poultry are generally more engaged with their clients and in herd health than those working with beef and sheep producers (ADAS, 2008, Ganter, 2008, Statham, 2012). One explanation for the difference in uptake by species and system is that some farm sectors have been heavily subsidised (sheep and beef) whilst others have not (pigs and poultry) with dairy cattle farming having some subsidy. Sheep farmers have been supported directly and indirectly by subsidy since the 1940s. Initially this subsidy ensured a minimum price per kg lamb produced, then an amount was paid per head of ewes in a flock (irrespective of their productivity) and currently sheep (and beef) farmers are given a single payment of approximately £208/hectare based on eligible land in good environmental and agricultural condition (Defra, 2012). The single payment scheme (SPS) comes under the EU's main agricultural subsidy scheme. In addition, from the 1950s to 1970s farmers had free or subsidised advice from agriculturalists and veterinary pathologists; both these subsidies have been reduced substantially in recent years (Woods, 2012).

The average flock size in the UK is 210 adult ewes (Defra, 2010), average lamb prices range from approximately £60 to £90 per carcase (EBLEX, 2012) and average lamb production ranges from 1 to 2 lambs per ewe, depending on farm location and sheep breed. Thus the gross income for a farmer with 200 ewes in 2010 was ∼£12–18,000 per annum; expenditures include rent, feed, labour and veterinary and medicine costs. Without the single farm payment most sheep farmers consider that they would not have sufficient income to support a family (personal communications).

Results from an independent survey (ADAS, 2008) of 2500 sheep producers indicated that 67% of sheep farmers only used their veterinarian for emergency treatment of sick sheep and that only 20% had regular contact with their veterinarian. This suggests that many sheep farmers do not value regular contact with their veterinarian. Frequent contact between farmer and veterinarian, together with a proactive relationship, could play a role in improving health and productivity on the farm (Jansen et al., 2010). Recently some initiatives have been set up to increase the involvement of veterinarians on sheep farms, these include subsidised attendance of veterinarians to set up flock health plans with sheep clients (ADAS, 2008). The benefit of these initiatives was difficult to evaluate due to the short time scale (one year) of the project, but tellingly, few farmers continued to maintain flock health visits once the subsidies were removed (Osler, 2009).

The sheep industry has several intractable disease issues e.g. anthelminthic resistance, a mean prevalence of lameness of 10% (Kaler and Green, 2008) and production inefficiencies, e.g. high perinatal lamb mortality, lack of adoption of genetic improvement. There is advice widely available to manage the above with, respectively, sustainable control of worms in sheep (Abbott et al., 2009), EBLEX better return programme manuals on management of lamb mortality and genetic improvement (EBLEX, 2012). One route by which this advice could be implemented would be by farmers liaising with their veterinarian to facilitate change in management to address these issues.

Although most sheep farmers have little contact with their veterinarian there is no information on why this is and whether understanding how sheep farmers perceive their veterinarian could improve health and productivity in their flock.

Focus groups are a well-established qualitative methodology used to explore and understand relatively new or complex issues where the aim is to understand beliefs that influence attitudes and behaviour of people (Kitzinger, 1995). They are widely used in various disciplines, e.g. sociology, psychology, education, political science and public health (Kitzinger, 1995) to collect data from several individuals simultaneously in a non-threatening environment, enabling individuals with shared experiences to discuss their perceptions, thoughts and opinions (Krueger and Casey, 2009). The use of qualitative methodologies is growing in veterinary research (Coe et al., 2007, Gunn et al., 2008, Christley and Perkins, 2010, Higgins et al., 2013) because an in-depth understanding of opinions and beliefs of farmers/owners is crucial to translating research into practice to improve health and productivity.

The current study used focus groups to explore sheep farmers’ beliefs and opinions of the role of their veterinarian currently and whether they perceived a role for veterinarians in providing advice to improve the health and productivity of their flock.

## 2 Materials and methods

### 2.1 Study design and selection criteria

Data collection was planned to continue until saturation of ideas/categories occurred (Morgan, 1997, Krueger and Casey, 2009). A total of 7 focus groups stratified by geographical region and farmer age (≤50 and >50) were carried out in England in 2010. There were two focus groups held in each of the north, centre and south east of England and one in the south of England. Inclusion criteria were male farmers producing lambs for meat with a flock size of at least 200 adult ewes.

An introductory letter describing the aims of the study and a pre-screen questionnaire (asking information on age, gender, flock size, other enterprises and level of veterinarian contact) was sent to 80 sheep farmers per region randomly selected by EBLEX (the levy body for beef and sheep farmers). Farmers were invited to participate in a focus group, interested farmers were asked to respond by completing the questionnaire. It was highlighted that the number of farmer places were limited. Travel expenses of £20 were offered to each participant.

From the pre-screen responses, 8–10 farmers per focus group that met the selection criteria with a range of age, flock sizes, other enterprises and level of veterinarian contact were purposively selected and invited to participate. A letter confirming the venue and some initial details of the focus group process was sent to them. A total of 45 farmers attended one of the seven focus groups. Details of the number of participants per focus group by age group, region and range of flock sizes are presented in Table 1.

**Table 1:** Number of farmers who attended each focus group by age, flock size and region.

Location	Age ≤ 50	Age > 50	Flock size (range)
Central England	5	10	200–800
North England	4	6	300–1500
South East England	6	8	200–1250
South England	6		250–1000

### 2.2 The focus group process

Ethical approval was obtained from the University of Warwick and all the farmers gave signed written consent after they had read an information sheet with details on the study objectives, data collection methods, audio recording and data confidentiality. The focus groups were conducted within a maximum 40 min drive of the participants’ farms. All the focus group discussions were guided by the same moderator (LEG) and observer (JK). The moderator facilitated the group discussions and the observer made notes of the discussion, seating plan of farmers, dealt with late-comers and arranged refreshments.

A discussion guide was developed by the authors and used by the moderator to facilitate discussion. The discussion guide was structured around two main areas of interest: (i) farmer use of veterinarians and how they perceive the veterinarian's role in improving health and productivity and (ii) current sources of information from which farmers updated their knowledge including the veterinarian's role in updating their knowledge. The moderator used prompts and questions to generate and facilitate discussion. To ensure robustness of the data various strategies were employed (Krefting, 1991, Krueger and Casey, 2009), these included expansion and rephrasing of questions and a summary of the discussion provided by the moderator to which farmers were invited to agree or expand. At the end of the discussion the moderator summarised the key discussion points and then farmers were given the opportunity to make any further comments if they felt an area had not been fully explored. Each focus group discussion lasted approximately 90 min.

### 2.3 Analysis of focus groups

Focus group recordings were transcribed and anonymised for analysis. The transcribed focus groups were analysed by JK using thematic analysis following the procedure by Braun and Clarke (2006). Analysis was inductive with themes driven from the data collected. Transcripts were first read and the content familiarised before coding. Coding was done in NVivo 9.0 (QSR International) and codes that summarised the meaning of text segments were used. Coded sections of the transcripts were then organised into preliminary themes. Transcripts were re-read and double-coded and discussed with LEG to maximise reliability. Constant comparison methods (Glaser and Strauss, 1967) were used to ensure that data that did not fit a most common dominant pattern were not ignored and also to assess across group saturation (Yardley, 2008). The data were interpreted with the focus on the study research questions.

## 3 Results

### 3.1 Characteristics of the farmers

There were 45 farmers that participated in the study; the median flock size was 350 (iqr: 300–600) (Table 1). A total of 12 farmers had only sheep on their farm and the remaining 33 had other enterprises which included one or more of cattle, pigs, hens, arable, holiday cottages, turkeys, agricultural business and mobile seed cleaning.

### 3.2 Thematic analysis

After the final coding there were 4 key themes: (a) current use of veterinarians, (b) barriers for proactively using veterinarians, (c) perceived benefits of proactively using veterinarians and (d) updating knowledge. These themes were similar across the regions and between the age groups. Themes and sub themes are described below.

### 3.3 Current use of their veterinarian

The majority of farmers had very little contact with their veterinarian and viewed the role of the veterinarian in their sheep flock as a fire-fighter (i.e. someone who they used in a crisis, this might be individual sheep that they could not lamb, but more often it was when several sheep had died from an unrecognised cause). Farmers considered that they knew their stock and their farm and the only time they would contact their veterinarian was when there was a major problem. The quotes below describe these views clearly. In the UK farmers refer to veterinarians as ‘vets’ we have amended all quotes to veterinarian (s) for an international audience.

“Veterinarians fire fight for a best description; they don’t do a lot of preventative work”

“We don’t have a lot to do with the veterinarian, only lambing or if we get trouble with worms and the wormer doesn’t work we take one for sampling and that. But otherwise we … we’ve learnt a bit over the years ourselves”

There were a few farmers that described that in order to get medicines they had to have contact with their veterinarian; and that was also their way of keeping in contact, e.g. as outlined by this farmer:

“We have to talk to a veterinarian every six months to be able to get medicines and stuff back, otherwise they just don’t let you have it, so that's one way of keeping in contact with them, actually”.

Farmers with cattle on their farms described using the veterinarians for advice for sheep ‘while they were on the farm’ attending the cattle. They said that they tend to ask any questions that they had on sheep during such visits and found that very useful. Farmers’ comments below, with some humour, describe these views clearly:

“We’re quite lucky because we’ve got the dairy, so we get regular vet visits to the dairy. So if ever we’ve got a problem or anything we just tend to chat to the vet when he's there sort of thing really”

“…when you have them with a cow or something and you change the subject onto sheep…. even vets can multitask.” <Laughter>”

Most farmers stated that they frequently used free telephone advice from their veterinarian. Farmers considered this a very useful route to obtain advice when needed. When they were asked if they would be prepared to pay for such telephone advice, there was a general view that this is something they expected from their veterinarian without paying for it.

“I try to phone him up or leave a message on his mobile to give me a bell [telephone call] when he's finished so we can have a ten minute chat about something…its good… I pick his brains on all sorts of different things”

“I don’t mind paying for an hour doing something, but I expect advice [on the telephone] for nothing”

A very few farmers described using their veterinarian/non-veterinary trained consultant frequently for advice. These farmers said that this input was very useful for improving health and productivity of their flocks. A comment on this is below

“I use my veterinarian quite a lot, for advice, specific things. I mean I was just thinking today, it's almost like … Venn diagrams with two big circles. And obviously you’ve got one over here with day-to-day welfare of sheep and that's the one-off treatments, and over here you’ve got year-long planning, flock plans, health plans and there's a bit in the middle where it obviously overlaps, for me anyway. And I use them for general advice.”

### 3.4 Factors that prevented farmers from using veterinarians for proactive flock health

There were many factors that farmers reported that influenced their decision on whether to use veterinarians proactively. These included their perception of a veterinarian's knowledge, lack of time, continuity of personnel and the economics of sheep farming.

#### 3.4.1 Knowledge

There was a general consensus among the farmers that there are not many veterinarians that are sheep specialists and so they do not consider veterinarians are able to make improvements in health and productivity in their flocks and hence they do not use the veterinarians proactively. The farmers’ discussion below highlights this:

“You’ve not actually got any specialist sheep veterinarians, really, have you?”

“I think you definitely need, like you say, a specialist in the job who's going round doing it, ‘cause if they picked anyone from ten veterinarians at our place, similar problem, never seen a sheep, ‘cause we never have anything to do with … I mean they treat them but they don’t specialise in ‘em like you said…”

Most farmers believed that veterinarians lacked a broad knowledge of sheep farming, especially non-disease related aspects, e.g. nutrition, genetics. In order to use a veterinarian's services more than they currently were, they wanted veterinarians to be knowledgeable in these areas. Comments below by farmers outline these views clearly

“I think you’d have to have a very good all-round knowledge of sheep farming right through, and I don’t know where the veterinarians going to acquire that without actually farming them as well. Very few know about grassland production as well as specific diseases and things. They’re good on the diseases but it's the whole … everything is so interlinked, isn’t it?”

“I doubt I would ask a veterinarian about EBVs (estimated breeding values). He wouldn’t know. I think I’d know a lot more about EBVs than any of my veterinarians”

“I would like my veterinarian to deliver into other areas which are well covered by other experts, nutrition, genetics, breeding”

Other people ‘yeah’.

#### 3.4.2 Lack of continuity of personnel and lack of time

Some farmers felt that lack of continuity with veterinarian's attending the farm limited their use of veterinarians whilst others commented that the rate of turnover of veterinarians in practices hampered their ability to start building trust and confidence.

“I think one of the problems with the vets as well is that it's difficult to get consistency because there's such a turnover of vets, in our practice in particular, and to get a really good sheep specialist. In the big veterinary practices there is a massive turnover of younger vets in them”

“Very important, continuity; they know me, they know my farm, they know what we’re trying to do, they know my problems”

A few farmers mentioned that veterinarian's rushed their visit and this prevented farmers from forming a trusting relationship with them. But a few also acknowledged that it was also sometimes farmers’ lack of time, either being unorganised or reporting back on the outcome of treatments to the veterinarian that is the problem.

“They probably haven’t got the time to go join us today, having a conversation or staying on the farm. We’ve got one veterinarian in particular, I don’t know whether his car is turbo-charged or whether it's jet propelled but he comes in, does the job and he's gone again. He's hardly got the chance to say …”

“I think we ought to be more organised with our veterinarians. I end up not finding the time because I’ve got quite a lot of other things to do, which probably sounds a little bit selfish but it's just the way I’m bred”

“I think to a certain degree farmers are at fault as well because we are very bad at feeding back to the veterinarian. He gives us something to treat an animal and it doesn’t work. We’re very bad at going back to him and saying, ‘Look, you need to change that product because it's no damn use, it's not working.”

#### 3.4.3 Economics of sheep farming

Most farmers believed that the economics of sheep farming is a major barrier for using veterinarians more regularly for advice or having more frequent contact with them. They mentioned that they could not afford veterinarians’ services because each sheep had little value (lambs about £90 at the time of the meetings, ewes about £120).

“I’m sure he could give us useful advice. It's just the economics of it and always has been, hasn’t it? The trouble with the job is that there's such a big, deep divide between the cost of having a veterinarian out and what the animal's worth at the end of the day”

When they further discussed this and were asked where they made money, most farmers mentioned the single payment scheme (SPS), many farmers believed that this was essential to sheep farmers and that they could not farm without it.

“It's a sad reflection, isn’t it, when the single payment scheme more or less equates to farm income last year”

“Ah … very! Very important [about single payment scheme]. I definitely don’t make enough that I can afford to put it away, which I know is what we all should be doing, but I don’t make enough money to do that. You should be able to. Just can’t really.”

When prompted, most farmers did not know where they made or lost money within the sheep enterprise and mentioned that their record keeping was not good, something they did not like to do but they should do more. The quotes below describe these views:

“I think record keeping is so important. I am always telling myself I’m bad at it, but I’m getting better … writing down everything that, every loss, all your losses, and what a ewe goes down with, so next year you can just … you’ve got to write it down, ‘cause you try and you forget like. I’ve done that”

“I do think it would be a very good and useful exercise to do [keeping records], but me actually doing it, I don’t think I’d do it, because the time you spend, I tend to think my time's better spent with the animals than actually in the office, and I hate office work…”

However, three farmers who said that they kept detailed accounts of their business also stated that they did not include the single payment scheme in their business plan. For them it was either taken away by the estate or owner of the land or they used it to making life easier or to re-invest in the farm as describe one of the farmers:

“… I put my single payment away, when I could afford it we used it for other things. For reinvestment and making life easier.’ I bought a Prattley race the first year, which made my life a lot easier!”

One estate farmer used a non-veterinary consultant. He viewed this as beneficial to improve flock health and production. He managed of a flock of ∼1500 ewes on a large estate with many enterprises and estimated that the consultant fees cost ∼£3000 per annum. He did not get the single farm payment, his flock was ‘fully’ recorded and he had to justify the income and expenditure from his flock to the estate accountants. This manager also highlighted that the consultant also gave support and credibility to the flock manager's decisions when explaining these to the estate manager (the flock manager's line manager).

### 3.5 Perceived benefits of using veterinarians proactively

Some farmers considered that only bad farmers need veterinary help.

“A veterinarian coming on regularly, that might help the farmer who's bloody useless to be honest, then they can pick up on all your problems, but most farmers are pretty good with their stock, generally speaking”

Others who had a trusting relationship with their veterinarian had confidence in their expertise and said that a veterinarian visiting the farm regularly provided an independent perspective.

“I’m sure the veterinarians just open people's eyes when they walk round the farm, to say, ‘I wouldn’t do that if it were me.”’

Another view was that having a veterinarian coming regularly on the farm help to get things done, having a deadline

“I think I’ll benefit; if I’ve got someone coming then I make sure those jobs are done. Myself, it's time is so precious, but if you’ve got deadlines you’ve got someone coming or visits, you get things done. And I think from that point of view it's a good discipline. It would be quite useful”

Some farmers considered that regular flock health monitoring was a way that veterinarians could add value to their flocks. But overall there was little clarity on how this model could work for sheep farmers. They said that routine flock health visits were mostly used in the dairy industry and veterinarians should instigate such monitoring for sheep farmers. After discussion there was agreement that small flocks could work together with a veterinarian and that they all could benefit financially.

“It's planning ahead, not fire fighting, because half the stuff we talk about is something that's ill today, we need to be preventing that. A bit like the dairy industry does, looking at nutrition at lambing time to help us with the colostrum, to stop the clostridial diseases that way, not just saying, I’m losing lambs with pasteurella, what am I going to do?”

“But I think the veterinarians have got to be the i nstigators of doing this sort of plan, like the dairy plan, how do we start it?”

“I feel that veterinarians are used very much by the dairy industry and an integral part, especially in the large ones, they’re always there, once a week visit and that sort of thing, and I think sheep could be treated in a similar way and I think although we’ve only got 400 ewes, they could be … if it was between a group of ten (sheep farmers) or something, going round, of similar type of sheep, it could be very useful having them come and just keeping you up to speed really.”

### 3.6 Updating knowledge

When farmers were asked how they updated their knowledge, some older farmers remembered free farm visits with advice on husbandry and grassland management and free post mortem examinations and visits from state veterinarians when disease outbreaks occurred. This advice was still considered useful for current farming (although 30+ years old). Many farmers listed many sources of information (most of them free) including farming magazines, newsletters from farming groups and veterinarians, EBLEX, Moredun, farming shows, animal health suppliers and the internet, also listed by farmers in Wassink et al. (2010a). Most farmers were of the view that there is enough information available to keep them updated. Some, but not all, farmers were sceptical of meetings organised by veterinarians; they were of the view that these meetings are generally sponsored by pharmaceutical companies and veterinarians could have a biased view.

“The veterinarians tend to get sponsorship for these sort of meetings, don’t they? They tend to have a biased view then a little bit because you get the whatever product they’re pushing at the time forced at you as well on the night”

“……vets are under pressure from the pharmaceutical companies to shift more and more of their products.”

## 4 Discussion and further commentary

This is the first paper to authors’ knowledge to explore sheep farmers’ opinions of current use of their veterinarian and whether sheep farmers view veterinarians as a route to add value to their farm business through flock health planning. There are few flock health schemes that survive unsubsidised (Osmond, 2009; personal communications) and it is not clear why this is the case and this paper sought to explore this issue from the farmers’ viewpoint.

A diverse range of male sheep farmers by age, flock size, type of sheep farming enterprise and geographical region were purposively selected to capture a range of opinions in the focus groups. Each opinion is qualitatively relevant and helps to understand how sheep farmers currently view the role of their veterinarian in the context of their farm. This qualitative approach is a valuable way to understand the diversity and depth of opinions of farmers on this topic (Yardley, 2008). We cannot quantify opinions, nor can we be confident that we have every opinion held by sheep farmers. Despite this, we consider that the approach used highlighted strong convergences within themes, theme saturation and similar opinions and has captured novel information that it is not possible to capture through quantitative questionnaire methodology. The aim and purpose of this type of qualitative research is to enhance understanding, enlarge insight and generate new hypotheses (Johnson, 1997, Yardley, 2008).

Combining the comments, one overarching impression is that the sheep farmers in this study viewed themselves as experts in sheep farming and in particular of their own farm. They considered sheep farming complex and that the years of experience that they had gave them a unique understanding of farming sheep. In general, they viewed themselves as the ‘best’ person to understand how to manage their sheep and most had a view that the veterinarian (or any other outsider) had a limited contribution to make to their overall flock management. They used the wide range of free information to cherry pick what might be appropriate for their farm. There was no consensus of one good source of information but there was some quality control e.g. specifically highlighted in our discussions were that sponsored veterinary meetings were viewed with caution and a few farmers considered that veterinarians ‘needed’ to sell pharmaceuticals and so lacked independence. The farmers considered that whilst there are similarities across sheep farms each farm is unique and farmers have found their own farm specific solutions to manage their flock on their farm. This idea of uniqueness and complexity came through with the farmers’ desire for veterinarians who were also sheep farmers and a suggestion that very few veterinarians could be sufficiently expert to advise them.

The one veterinarian role accepted by all farmers in the study was to provide help during an unknown disease event that caused raised levels of morbidity or mortality. The farmers described this as ‘fire-fighting’ and viewed it as an economically costly event (animals died and they had to pay for veterinary time and pharmaceuticals) but one where veterinarians were uniquely qualified to assist. Once the cause of the disease and management was known the veterinarian had no further role to play, and subsequent outbreaks/endemic levels of the same disease would be managed by the farmer with minimal input from their veterinarian.

Outside this major role, most farmers in the focus groups considered that their veterinarian played a role in advising them on other diseases that occurred in their flock that were not emergencies. They generally obtained this advice free of charge either by telephone, visiting the practice premises or discussing their sheep when the veterinarian was visiting the farm for another purpose, such as tuberculosis testing of cattle. This was common practice and getting free advice was considered a reasonable behaviour.

When farmers were asked to consider using their veterinarian in an advisory role in flock health there was some acceptance of a useful role by a few farmers, with the veterinarians providing an ‘outside eye’. Generally, however, there was little understanding on how flock health planning might be done and farmers looked to veterinarians to provide a model. There were several barriers raised, most farmers did not consider that their veterinarian knew sufficient about farming sheep and non-disease aspects of production in general and about their flock and farm specifically and so was not in a position to offer advice. Once again this is the idea that sheep farming is very complex and each farm is unique. This could be a major hindrance to developing flock health plans, particularly in the absence of any productivity and health records which does indeed make a farm complex because of the unknown.

In addition, many sheep farmers had farmed sheep for the whole of their life as had their fathers and grandfathers and they wanted a veterinarian that they had known for many years. Farm animal practice in the UK is changing (Lowe, 2009), farm practices are merging and so there are more veterinarians per practice and veterinarians now move between practices gaining experience in several locations when they first qualify. It is increasingly unlikely that sheep farmers who have a veterinary visit once or twice per year will know the veterinarian visiting their farm. This difference between veterinarian and farmer generation time is likely to continue and our results indicate that some thought needs to be given on how to improve veterinarian farmer interactions if turn-over of veterinarians continues to be high. The issues highlighted in the current study of lack of interpersonal trust, confidence and consistency are also central to public trust in healthcare (Gidman et al., 2012) and congruent with the social theory of trust developed by Luhmann (1997). Luhmann's theory highlights the close and complex relationship that exists between trust, confidence and familiarity. Confidence can stimulate an individual's activities in situations of uncertainty and trust develops with familiarity, and familiarity is used by an individual to calculate risk. Thus farmers are more likely to develop trust in veterinarians as their experience of their veterinarian increases.

Where farmers had a positive view that their veterinarian could have wider input in the farm it was generally because the veterinarian had been proactive and offered health care packages, e.g. to monitor helminths to improve control. In these cases, the veterinarians were usually charging a low rate to gain uptake from farmers and it was not clear that farmers would have paid the full economic cost of the programmes.

This leads to another barrier highlighted by farmers, the cost of veterinary time–veterinary costs were considered to be high. However, when farmers were asked about the economics of their farm and whether they made money from their sheep, most did not record where or how they gained and lost income but considered that the single payment scheme was the only reason that they could continue to farm sheep. This guaranteed SPS income is ‘topped up’ by income from selling lambs.

A negative result of this financial support is that it reduces sheep farmers need to keep records and understand where flock income and expenditure arise. Only three farmers in 45 kept records which enabled them to evaluate the economics of their flock and identify where they gained and lost income. These are the same records that can be used to make decisions on flock management to improve income. Without these a flock health plan cannot be instigated because there are no baseline measurements to use to monitor the impact of management changes. If sheep farmers are unaware of their income, or do not link changes in flock management to increased income then many of the recommended health (Wassink et al., 2010b) and management (EBLEX, 2012) improvements promoted based on increased income will not be adopted.

The one flock manager with 1500 sheep who used a non-veterinary consultant was quite different from the other 44 farmers in the study who were responsible to no one for the management decisions they took. The ‘fully’ recorded large flock and external consultant model is similar to that used by the dairy and pig sectors but our results suggest it would not be appropriate for most sheep farmers currently.

Most farmers in the focus groups could not conceive paying a rate per sheep in the flock for external advice. One issue was that sheep flocks are generally small (less than 400 ewes) and so a rate per ewe that would provide e.g. four visits per year (£2000 was considered excessive). As the farmers highlighted, there are few role models of sheep farmers using a veterinarian as an advisor where the veterinarian adds to the farm profitability so this is a new concept for many of them.

Our results suggest that there is an impasse, veterinary input would cost money but sheep farmers currently, generally, do not consider that they would see financial benefit from this cost, partly because they do not keep records that could be used to measure the difference an advisor could make. Lack of production records and the view of SPS money as a subsidy for sheep farming might also explain the slow uptake of other new developments in the industry. The price of lamb in the UK in 2010 was double that of 2006 (EBLEX, 2012). Whilst some expenditure also increased, e.g. the price of purchased feed, sheep farmers could/should have had an increased income from 2006 to 2010. There has been some commentary that sheep farmers should/could have improved their flock with this extra income (EBLEX, Better Returns Programme) but few have done so.

There is a view that although the SPS secures a physically attractive and environmentally cleaner countryside that it reduces the sheep industry's competitiveness. The SPS is not likely to be abolished in the near future (Defra, 2012) and it is not clear that removing subsidies would lead to increased use of veterinarians for proactive flock health; for example, in New Zealand where subsidies have not been given since the 1980s, sheep farmers with <800 ewes still rarely use their veterinarian (personal observations, the authors). English sheep farmers are also not reliant solely on income from their flocks so they might be less likely to become production efficient without some other incentive. Including production and endemic disease monitoring in the SPS has been mooted (FAWC, 2011). This would certainly give a baseline set of data that farmers and veterinarians could work with to develop flock health plans and to monitor improvements in the absence of direct economic incentives to the farmer.

## 5 Conclusions

In conclusion, there were strong convergences within themes, theme saturation and similar opinions that emerged from farmers with a range of flock sizes, ages and from different geographical locations. The majority of sheep farmers who participated in the study considered their veterinarian as a ‘fire-fighter’. They could not easily conceive that veterinarians had a major role to play in flock health planning because of their lack of expertise in sheep husbandry and farming in general and of their sheep farm in particular. In addition, sheep farmers considered paying a veterinarian for the time required for sheep health planning to be too great a cost, however, they did not know where they made and lost money in their flock because they relied heavily on income from the single payment scheme. We consider that this is an impasse: flock health planning will not be common practice until veterinarians demonstrate greater expertise in sheep health, husbandry and farming and provide a model of how flock health planning would be financially beneficial and also develop a trusting relationship with their clients. In addition, until sheep farmers keep sufficient records and financial accounts to understand where they gain and lose money they will not appreciate whether and how flock health planning could benefit their flock and their income. Including the requirement to keep health and production records as part of SPS and so provide baseline records might lead to flock health planning being adopted at a faster rate and so develop the UK sheep industry and make it more environmentally sustainable by reducing waste from disease and low productivity.
